# The Psychology of Failure and Success

**Published:** 1908-08-22

**Authors:** T. Claye Shaw

**Affiliations:** Lecturer on Psychological Medicine at St. Bartholomew's Hospital.


### Hospital Clinics.

### THE PSYCHOLOGY OF FAILURE AND SUCCESS.

By T. CLAYE SHAW, M.D., F.R.C.P., Lecturer on Psychological Medicine at St. Bartholomew's Hospital.

(Abstract of a Lecture delivered at the Medical Graduates' College and Polyclinic.)

The authorities of this institution have selected out of several subjects which I submitted to them, that of "The Psychology of Success." I will, therefore proceed, with your permission, to say what I have to put forward on that question.

The wise man will do well to analyse the processes by which he has succeeded or has failed; to note whether he was of necessity led to one result or the other by circumstances which he could not control, or whether he was so able to steer his course that at last, as the French say, he arrived. In this process of introspection he will recognise that fortuitous occurrences, of which he could have had no prescience, acted unexpectedly; but all these introspections, which no outside observer can more than imperfectly estimate, show that there is really a Psychology of Success, an exploitation of an existing basis capable of deflection and development by the actor in the little drama of his own life.

But though the outsider can have but a partial insight into the causes which have conduced to the success of another, his observations may be an important complement to the introspection of the protagonist. Our knowledge of the psychology of success is then a fusion of internal and external factors: and as the internal factor cannot always be analysed, while judgment of the external may be incorrect, it is manifest that a complete account of success in any definite case is not always practicable, though there may be a general consensus as to the main constants.

### Good and Bad Luck.

It is in many cases the occasional intervention of unintentional accidents, as they must be termed, which makes the difference between luck and success. We talk of good or bad luck, as an ancient would believe in his gods; but after all it is imagination which is at work when we account for results that we cannot trace out in their happenings by what is called chance. The usual idea of luck is that something favours or acts against you which you had no right to expect or reckon on. A man who stays at home all day, and can always be relied on to be at a certain place, gets patients; the man who *happens* to be in when the telephone summons him to go to a good patient is said to be lucky. He who backed the outsider for the Derby and won is said to be lucky, but if it is meant that a special Providence intervened on his behalf, then luck means a miracle, an alteration in the usual sequence of cause and effect.

Who is there who does not at times say, "as for the rest I must trust to luck;" or, if he fails, "just my luck." Instead he ought to say, "I must get to know as much as possible about the conditions, and leave nothing out which may lead to a different result from the one I want." Nobody wins when he ought not to do, though he may win when he does not expect to.

To be lucky is to succeed when you do not expect it, when as far as you yourself are concerned you do not deserve to, when you have unconsciously tumbled into the path which leads to success. To be unlucky is to find yourself placed in circumstances which are against a favourable issue, but are a necessary result of causes, some of which were either ignored or were not valid for your consciousness.

There must always be processes which are permissible to some, and yet positively criminal to others. For instance, betting on an issue is as a rule full of uncertainties; it is, however, allowable for those who find recreation in it, and are able to afford it, but it is a criminal thing in those who cannot afford to risk their money. To gamble is to hypothecate success, to override responsibility; it is rarely legitimate morally and ethically, and is at times actually illegal. As a fact there are few acts deliberately entered upon which are not to some extent a gamble, though the chances of impediment may be so small as to be rightly eliminable.

The man who elaborates a system and stands or falls by it is not a gambler; he is as legitimately entitled to his results as he who conducts any other form of business, and he shows that he is not a gambler because if his system fails he either corrects it or gives it up altogether. The desire of excitement and the desire of wealth are the chief psychological factors in gambling. Take away the first, and you find the fool; fool, because he ought to know that in striving to get hold of a large result for which he is giving no equivalent he is taking a course the outcome of which he cannot possibly predict.

### An Analysis of Success.

Success may be defined as the realisation of what is willed or wanted. It is the tomb of energy, the collapse of strife, and the satisfied goal of ambition. Too often success is made the end, instead of merely the beginning; the expenditure of force in the production has been too great, and the result, unable to nourish itself, dies from inanition and makes no progress. At times an accident, though as a rule the fruition of patience and well directed energy, it is often capricious and may elude the grasp of him who thinks he has secured it, and it is just this expectant uncertainty which stimulates the energy of those who find that they have attained a phantom, and that it is not the sought for object that has projected the shadow.

So many excuses are made for failure! Either the moment was not propitious, or interest in the matter had waned, or the markets had changed, or any reason rather than the right one, namely, that the thing could be done without or had missed fire at the critical moment. The public may know what it wants, or there may be an " is wanted " quite independently of what this same public thinks. But he who waits for a demand may find the supply remaining idle on his hands; the thing to do is to create the demand, and then if possible to corner the supply. Now, in business, this is much easier than in a profession, partly because the business man is less hampered by etiquette.

Success may really be a failure, because having been once achieved it may stop a man from proceeding to a higher development; such success seals up adventure and limits discovery. Success often comes from the most unlikely materials, because we cannot always predict just what is wanting to make a thing a success; it may be a sudden presentation or idea, or an opportunity, or some unexpected combination of circumstances. The failure of one individual may mean the success of another by the withdrawal of opposing impediments to the success of a plan; and even a purposed failure may be the cloaked means of success, as when a man goes bankrupt, and then rises from the ashes freed from encumbrances and ready to soar to the empyrean.

The usual estimates of success are expressed in terms of £ s. d., or of social position, or in self-satisfaction, and this objectivity or subjectivity may be taken as a fair exponent of what happens, though true appreciation of the result is not always reached. Want of contentment may even turn success into failure. One sees in the reluctance of men to drop their work after compulsory retirement at a comparatively early age the same spirit of desire for the following up of a so far successful career. Men talk of the *ennui* of having nothing to do, but it is scarcely complimentary to the man himself to think that he has no resources for occupation other than the continued monotonous reflex of what has been his life's work. Every intelligent man ought to be able to fill up his leisure by occupation derived from the side chains of his former duties. It makes one almost suspect that men really shirk the idea of dropping the so-called work and the contemplation of looking round for fresh fields.

### The Element of Hardness.

One finds that most very successful men have been those who knew how to delegate to others much of what had to be done. I admire such men; this power denotes the absence of jealousy and the possession of the intelligence to see that whilst it is really easier to fill one's time by work which has become a second nature, it is bad in principle, because the time is wasted which might more profitably be employed for new developments. I know some men who are voted hard taskmasters because they place on the shoulders of their subordinates much of the work which is their own function; but I admire those men, because I see how they are laying the foundations of success by employing these docile donkey-engines.

*Apropos* of these remarks we note that some of the most important parts of the mental content which makes for success are cruelty, sacrifice of others, and deceit. Well, what of that? the drama of Life involves all these. If the attainment of one object, whatever that object may be, is the equivalent of success, it means that obstacles must be ruthlessly overthrown, opposing feelings and sensibilities trampled on if they stand in the way; hence successful men are strong men. Did Napoleon or von Moltke consider lives of men at Austerlitz or Gravelotte? Do governments consider the feelings and the very means of existence of minorities? We all profess to admire Virtue, Charity, Courtesy to others; but as often as not virtue means incapacity, the man who invokes charity has probably an axe to grind, whilst courtesy is frequently an alias for the external gilding of a very bitter pill.

What is wanted to ensure success is above all things Courage. How many chances are thrown away because men lack courage, preferring the charted course of mediocrity to the risk and trouble of striking out a path for themselves! The man who courts success cannot afford to tread the path of those who are safe in their mediocrity; he must use them as stepping stones, which implies that he must put them off the scent of his own aspirations lest by chance he should arouse their jealousy, and find them pulling him back into their ranks.

### Success in Medicine.

If we turn to our own profession and try to appraise the qualities which have been associated with, if they have not actually led to, a successful career—*cela donne furieusement à penser*. I have often observed how very uninteresting in private life are many persons of great prominence in literature and art; yes, and in science too. The reason is obvious; nowadays there is such competition for new ideas that writers cannot afford to give clues of possible success to others. And so men who have achieved or are well on the road to success will sit silently by absorbing all that is being said or done around them; taking everything, but giving nothing. I remember a great society entertainer, whose sallies provoked peals of laughter from his audiences; but privately he was a most tiresome person, quickly assimilating any atmosphere in which he happened to be placed or had thrown himself into, but conspicuous by a marked absence of reciprocity. Does the teacher or lecturer live, who gives all his best to his pupils? The latter may know all that he has taught them, but he has generally kept something up his sleeve.

Whatever knowledge a man may possess he must be able to make available if he is to be a recognised success. Of course, to be an actual success does not postulate publicity, but to be a convertible success the environment, *i.e.* the public, must know where to find what it wants; and this can only be done, speaking frankly, by advertising. There are scores of men in our profession who are equally qualified to succeed, but the majority of whom lack something which makes the public seek them out and employ them; and that something is usually that they are not known.

### The Uses of Advertisement.

For how indeed can the recognition of professional merit be expected unless it is known to exist, and the public be made to believe in it? The only way to get at the public is to advertise; but this has to be done carefully, and the legitimate paths for it are few and very jealously guarded. Perhaps there is more laxity in considering the bounds of legitimate advertising than was permitted not very long ago; and much is to be said for taking the public into professional confidence. The success of quackery depends on the shameless and lying advocacy of certain compounds which are prominently displayed before the public, which is only too apt to believe what is boldly asserted and is not contradicted. The public has probably a right to know the truth about what is openly published, and therefore, we must not pass too hasty judgments on those who, in the interests of truth, and, admittedly, of personal prominence, undertake to instruct the lay press and to correct vulgar errors.

It is easy to mention great scientific epochs, which won immediate distinction for their exploiters, but even here the publication of the various discoveries in the lay press must have been the source of the addition of a material to what was a nominal success. On the other hand, material success has come to many whose purely scientific claims have been *nil*, but who have influenced their surroundings by such means as a strong personality, prominence on public platforms, and various devices for engaging public attention.

### The Four Essentials.

But in whatever way success is achieved there are four mental necessities: a clear view of the end, a judicious indifference to the sentiments aroused by the sweeping away of obstacles, an indomitable energy, and the power to resist the temptation to remain on the soporific plain of mediocrity.

I have often wondered what would happen if men could live their lives again, retaining their experiences of mistakes and successes with a fair knowledge of the causes that led to them. It might be that the lines of former success would not be available because others would see what was being done and would checkmate it. Certainly some failures would be turned into successes, because former pitfalls could be avoided. On the whole it would be a tame world; things would be so certain that the interest attached to what we now call chance would not exist; the springs of hope would be cut off at their source, because definite knowledge would lead to emotional extinction. Energy would not be so necessary, for if quiet routine would insure certainty why not calmly await the issue? It seems only right that everyone should have his opportunity of proving his own intrinsic merit, and not be handicapped by the competition of those who have previous knowledge of the course.

